# Current practice of placental cord insertion documentation in Australia – A sonographer survey

**DOI:** 10.1002/ajum.12360

**Published:** 2023-07-26

**Authors:** Samantha Ward, Zhonghua Sun, Sharon Maresse

**Affiliations:** ^1^ Discipline of Medical Radiation Science, Curtin Medical School Curtin University Perth Western Australia Australia

**Keywords:** eccentric, marginal, placental cord insertion, protocol, sonographer, ultrasound, vasa praevia, velamentous

## Abstract

**Introduction:**

During pregnancy, the umbilical cord attaches to the placenta in a central, eccentric, marginal or velamentous location. Maternal and fetal complications are associated with marginal and velamentous cord insertions, the most clinically significant being perinatal mortality due to undiagnosed vasa praevia. Current literature describes a wide variation regarding regulation of placental cord insertion (PCI) documentation during antenatal ultrasound examinations. This prospective cross‐sectional study aimed to assess the current practice of antenatal PCI documentation in Australia.

**Methods:**

Members of the Australian Sonographer Accreditation Registry were invited to participate in an online survey which was distributed between February and March 2022.

**Results:**

Four hundred ninety sonographers met the inclusion criteria for the study of which 330 (67.3%) have more than 10 years' experience as a sonographer and 375 (76.5%) are employed primarily in a public or private setting offering general ultrasound. Most respondents (89.6%) indicated documentation of the PCI site is departmental protocol at the second trimester anatomy scan (17–22 weeks gestation), but PCI documentation is protocol in less than 50% of other obstetric ultrasound examinations listed in the survey. The PCI site is included in the formal ultrasound report at a rate significantly less than inclusion in the departmental protocol and the sonographer's worksheet.

**Conclusions:**

Considering the potential maternal and fetal complications associated with abnormal PCI and the ease at which the PCI site is identified in the first and second trimesters, we believe that standard inclusion of the PCI site in departmental protocol and in the formal ultrasound report from 11 weeks gestation, regardless of whether it is normal or abnormal, would prove invaluable.

## Background

### Clinical significance of the placental cord insertion (PCI) site

The umbilical cord inserts into the placenta in a central, eccentric, marginal or velamentous fashion.^1^, ^2^, ^3^, ^4^, ^5^, ^6^ Marginal cord insertion (MCI) has been variously characterised in the literature with definitions cited as <2.5 cm,^7^ <2 cm,^2^, ^5^, ^8^ and ≤1 cm from the placental edge.^6^, ^9^, ^10^, ^11^ Eccentric cord insertion is most commonly defined as a lateral insertion >2 cm from the placental edge.^12^ Velamentous cord insertion (VCI) occurs when the cord does not enter the placenta directly, rather it inserts onto the chorioamniotic membranes at a varying distance from the placental edge.^7^, ^13^, ^14^

Central and eccentric cord insertions are not associated with adverse pregnancy outcome, so they are collectively normal.^3^, ^15^ Marginal and velamentous PCI sites are abnormal and are associated with a variety of obstetric complications^1^, ^5^, ^7^, ^8^, ^16^, ^17^, ^18^, ^19^, ^20^, ^21^ which include but are not limited to those listed in Table 1.

**Table 1:** Obstetric complications associated with marginal and velamentous cord insertion.

Associated complication	Marginal cord insertion	Odds ratio	Velamentous cord insertion	Odds ratio
Vasa praevia			✓^14^, ^17^, ^22^, ^23^, ^24^, ^25^, ^26^	65.1^27^
Fetal/perinatal mortality	✓^28^	0.97^7^	✓^5^, ^7^, ^8^, ^13^, ^16^, ^22^, ^25^, ^28^, ^29^, ^30^, ^31^	2.14^7^
Low birth weight	✓^1^, ^3^, ^5^, ^7^, ^8^, ^10^, ^16^, ^18^, ^20^, ^21^, ^28^, ^32^, ^33^	1.24^7^ (birth weight <10th centile)	✓^1^, ^2^, ^5^, ^8^, ^13^, ^16^, ^18^, ^22^, ^28^, ^29^, ^33^, ^34^, ^35^, ^36^	1.88^7^ (birth weight <10th centile)
Preterm birth	✓^5^, ^7^, ^8^, ^16^, ^18^, ^21^, ^28^, ^32^, ^37^	1.28^7^	✓^5^, ^7^, ^8^, ^13^, ^16^, ^18^, ^21^, ^22^, ^23^, ^25^, ^28^, ^30^, ^33^, ^34^, ^35^	2.03^7^
Caesarean delivery	✓^1^, ^5^, ^7^, ^18^, ^28^, ^37^	1.19^7^	✓^1^, ^5^, ^7^, ^16^, ^18^, ^28^, ^34^	1.11^7^
PPH	✓^8^	1.07^20^ (PPH > 500 mL)	✓^8^, ^13^, ^20^, ^29^	1.62^20^ (PPH > 500 mL)
Manual removal of placenta	✓^1^, ^8^, ^21^	1.16^20^	✓^1^, ^8^, ^13^, ^20^, ^23^, ^29^	5.21^20^
Placental abruption	✓^7^, ^8^, ^18^, ^32^	1.48^7^	✓^2^, ^7^, ^8^, ^13^, ^16^, ^18^, ^22^, ^25^, ^30^, ^31^	2.6^7^
Low apgars	✓^16^	1.02^7^	✓^2^, ^7^, ^8^, ^13^, ^16^, ^22^, ^25^, ^28^, ^30^, ^31^, ^35^	1.87^7^
Neonatal intensive care admission	✓^5^, ^8^, ^16^, ^32^	1.29^7^	✓^5^, ^8^, ^25^, ^34^	1.83^7^
Intrapartum heart rate abnormalities	✓^1^		✓^1^, ^2^, ^36^	1.59^38^

PPH, postpartum haemorrhage.

Current literature documents the incidence of the PCI site in singleton pregnancies as >90% for normal insertion,^7^ 4.2%–11.3% for marginal insertion^2^, ^3^, ^5^, ^6^, ^7^, ^8^, ^10^, ^11^, ^15^, ^16^, ^18^, ^20^, ^32^, ^37^, ^39^ and 0.4%–2.4% for velamentous insertion.^2^, ^3^, ^5^, ^6^, ^7^, ^8^, ^10^, ^11^, ^13^, ^15^, ^16^, ^17^, ^18^, ^20^, ^22^, ^32^, ^34^, ^35^, ^39^, ^40^, ^41^ The diversity in the reported frequency of abnormal PCI may be due to the variety of definitions of MCI and whether the PCI site was measured on antenatal ultrasound or on pathologic examination.^10^

Whilst the current literature advocates antenatal ultrasound assessment of the PCI,^10^, ^25^ to our knowledge, no studies have been performed to determine how frequently ultrasound localisation of the PCI translates into formal documentation of the PCI site. This study assessed the extent to which the PCI is included in the departmental protocol, the sonographer worksheet and in the formal report during obstetric ultrasound examinations in Australia.

### Ultrasound of the PCI

**Figure 1:**
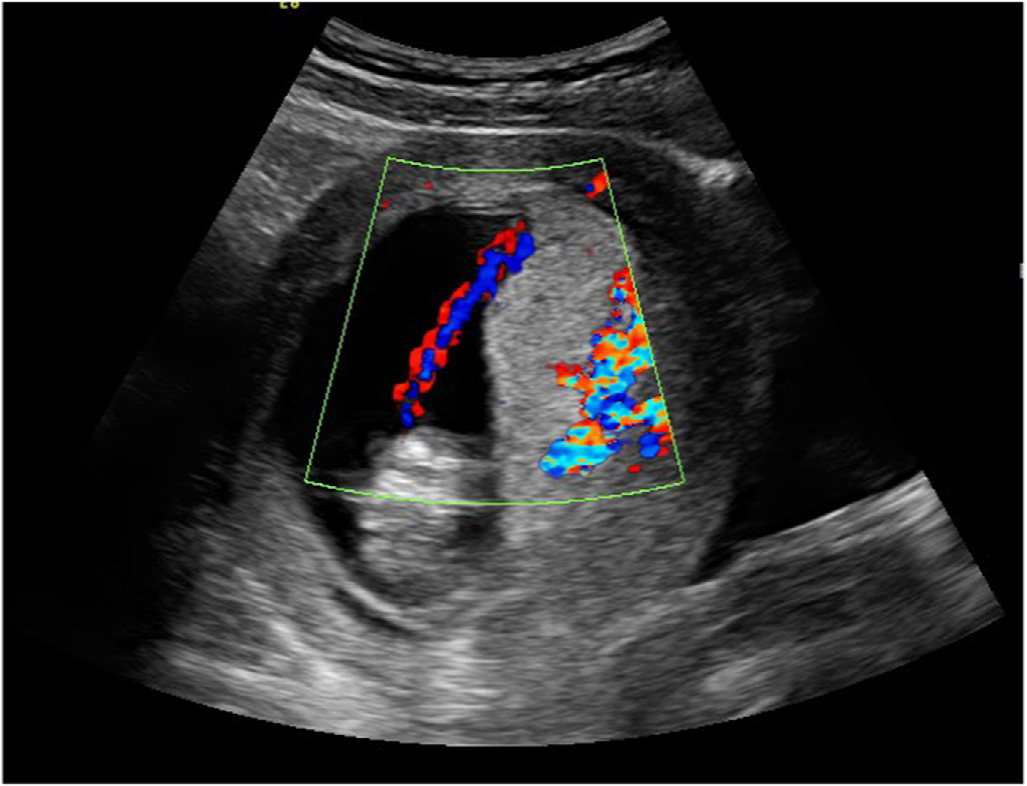
Marginal placental cord insertion in first trimester.

**Figure 2:**
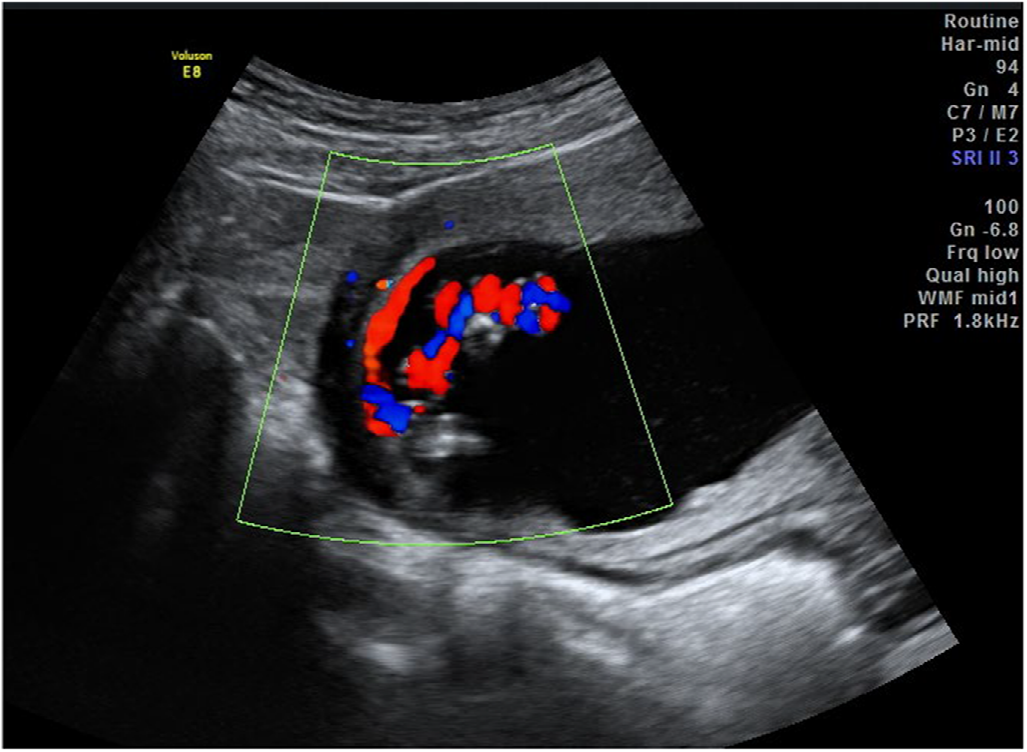
Velamentous cord insertion in first trimester, longitudinal view.

**Figure 3:**
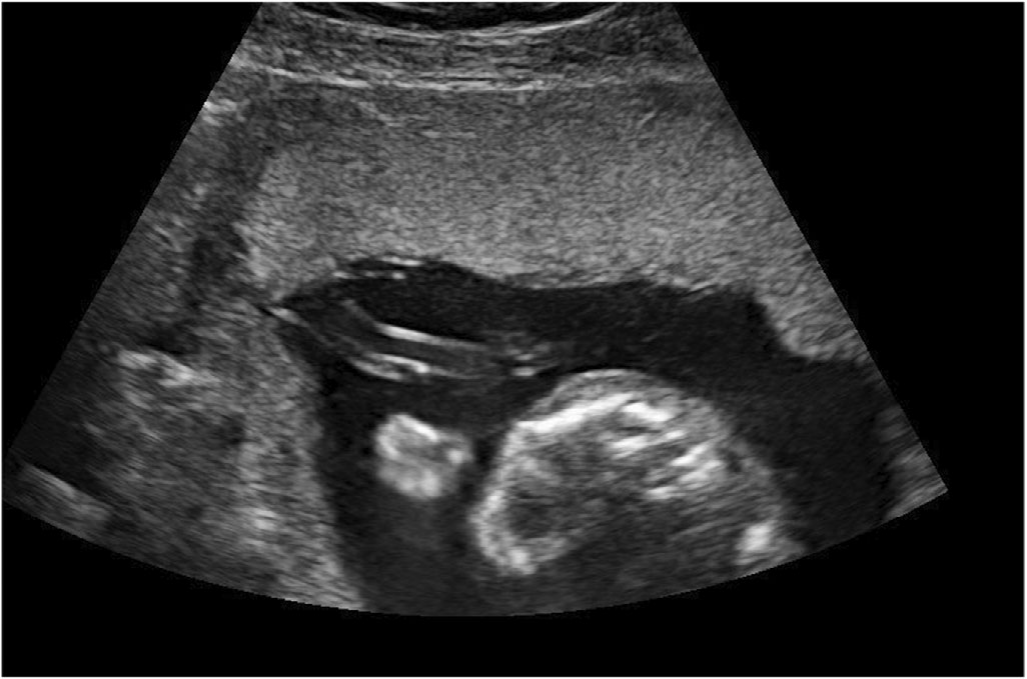
Marginal placental cord insertion in second trimester, longitudinal view.

**Figure 4:**
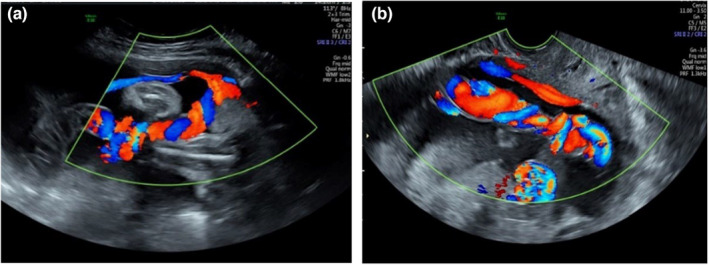
Velamentous placental cord insertion in second trimester. (a) Transabdominal view (b) Associated vasa praevia demonstrated on transvaginal view.

**Figure 5:**
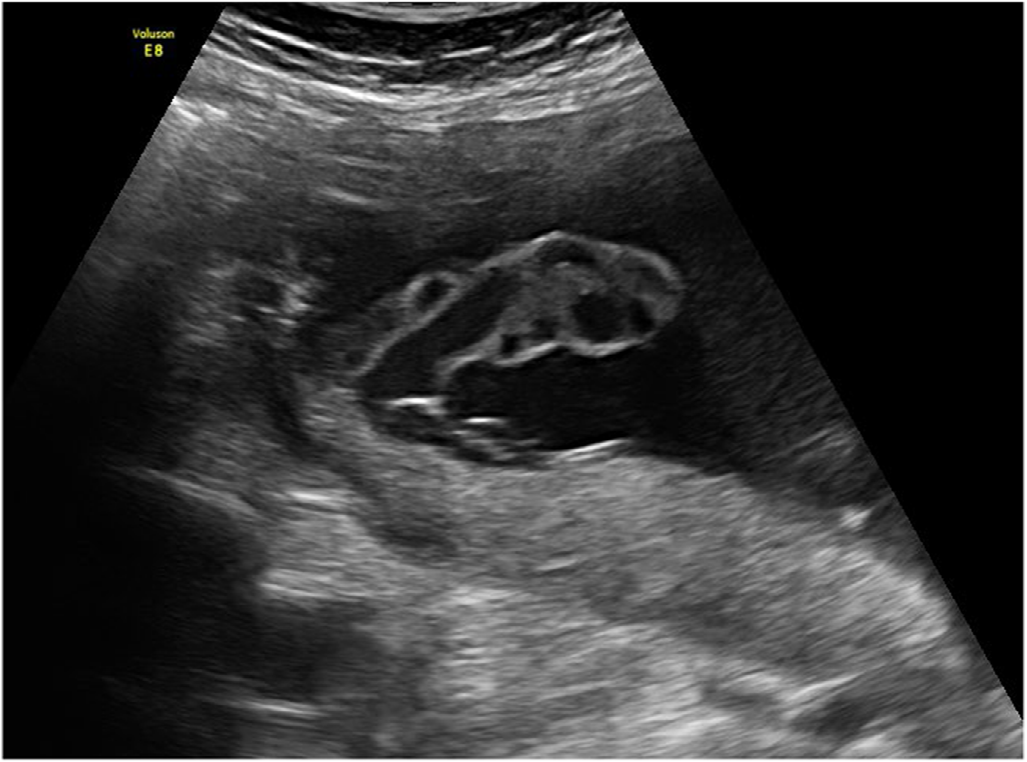
Third trimester marginal placental cord insertion, longitudinal image.

**Figure 6:**
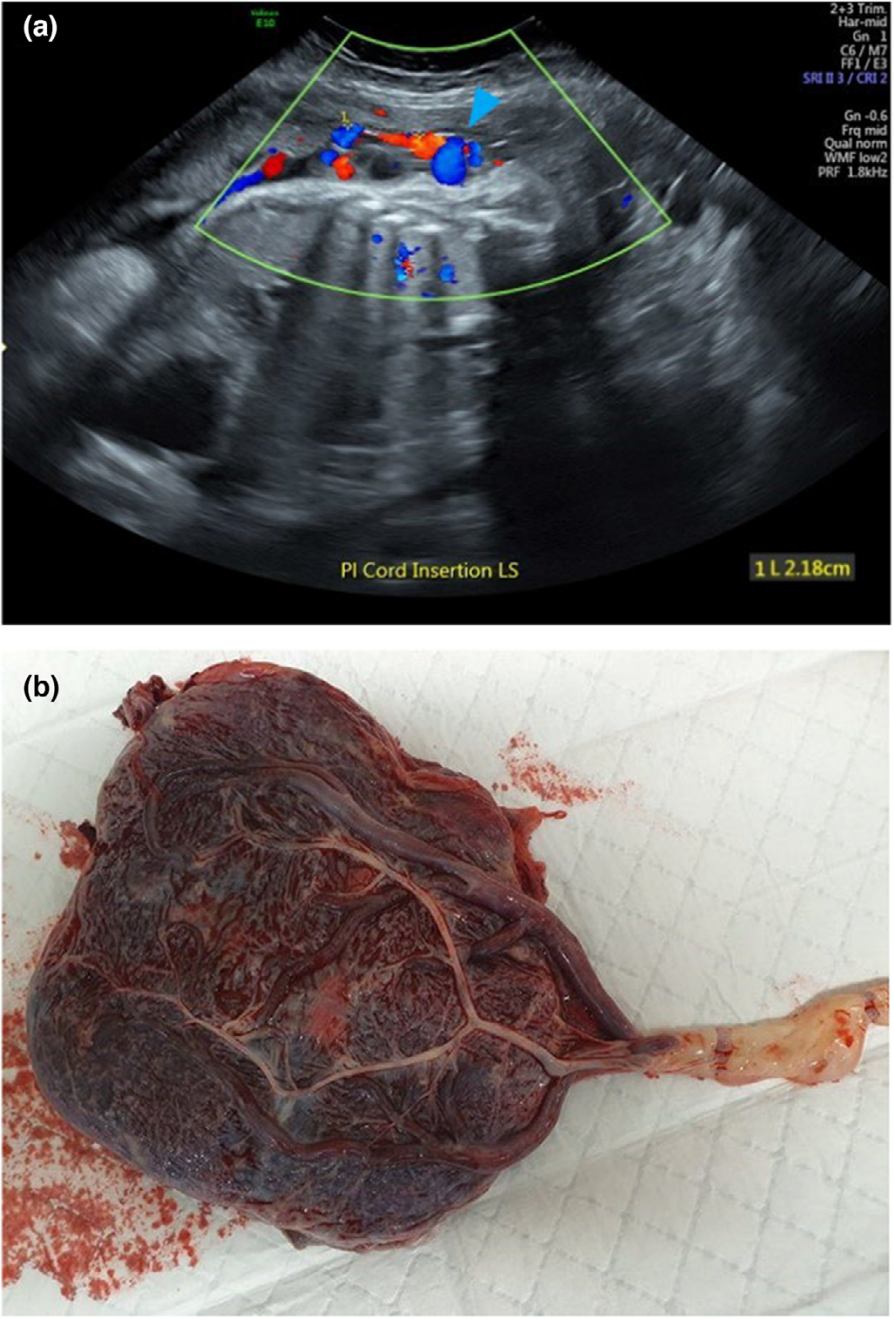
(a) Velamentous placental cord insertion in third trimester and (b) the placenta after delivery.

It is widely recognised that the PCI site can be readily identified on prenatal ultrasound.^11^, ^15^, ^16^, ^35^, ^42^, ^43^ In a study by Sepulveda *et al*.,^35^ colour Doppler ultrasound was routinely used during second and third trimester ultrasound and the PCI site was identified in 825/832 (99%) cases. Hasegawa *et al*. were able to demonstrate the PCI at the routine anatomy scan in 97.7% of cases.^8^ Several authors suggest documenting the PCI site as early as the 11‐ to 14‐week ultrasound^1^, ^31^, ^42^, ^44^ whilst others advocate routinely visualising the PCI site during every ultrasound examination performed.^10^, ^25^ Our experience has shown ease of documentation of the PCI site at all gestations in most cases (Figures 1, 2, 3, 4, 5, 6).

## Materials and methods

### Study design

This study was approved by the Curtin University Human Research Ethics Committee (HRE2021‐0629). An Australian Sonographer Accreditation Registry (ASAR) board‐approved online survey was created using Qualtrics (Qualtrics LLC, Provo, Utah, USA) and the survey link was distributed *via* email to members of the ASAR who have agreed to be contacted regarding research (n = 7353). The survey was available from 23 February 2022 to 23 March 2022 and informed consent was obtained before participants commenced the survey (see Appendix S1). Survey respondents who declined to participate and those who do not perform obstetric ultrasound were excluded from the study. Participant demographics including practical experience and the primary employment setting was obtained (Appendix S1). Quantitative and qualitative data were collected utilising open and closed responses with the following objectives:

1. Assess frequency of inclusion of the PCI site in departmental ultrasound protocols. Respondents were asked to indicate whether it is departmental protocol for the PCI site to be documented in their primary place of employment during various ultrasound examinations performed in pregnancy. We analysed the data further to determine whether there is a correlation between protocols within a general ultrasound and obstetric and gynaecological (O&G) ultrasound setting.
2. Establish the extent to which the PCI is currently documented:
  - By sonographers on the sonographer worksheet. Our study aimed to determine the frequency of inclusion of the PCI site on the sonographer's worksheet in the absence of departmental protocol. We explored this further to determine whether there is any correlation between a sonographers clinical experience, their primary workplace setting and their documentation of the PCI site on their worksheet.
  - By the radiologist/sonologist in the formal ultrasound report. Sonographers were asked at which obstetric ultrasound examinations the PCI site is included in the formal report in their primary place of employment.
  - During follow‐up ultrasound examinations. We asked sonographers whether they would reassess the PCI site after the second trimester anatomy scan.
3. Explore sonographers' opinions regarding the value of PCI documentation. We asked sonographers for their opinion on the value of documenting the PCI site with respect to (i) how important sonographers feel it is to document the PCI at every ultrasound examination and (ii) whether sonographers feel they can make a significant difference to maternal and fetal outcome by documenting the PCI site. The data were further analysed to determine whether there is any correlation between a sonographer's opinions and their years of experience and primary place of employment.

### Sample size

For a population size of 7353 using a confidence interval of 95% and a margin of error of 5% the estimated sample size is a minimum of 366. The survey elicited 534 responses (response rate 7.1%). Of these 534 responses, five (0.94%) declined to participate and 39 (7.3%) do not perform obstetric ultrasound, and were therefore excluded from the study, leaving a sample size of 490.

### Data analysis

Survey responses were analysed using IBM SPSS Statistics v26 (IBM Corporation, Armonk, NY, USA) and Microsoft Excel v2209 (Microsoft Corporation, Redmond, Washington, USA). Quantitative data were evaluated using descriptive statistics (frequency and/or percentage). The Pearson chi‐square test was used to determine the correlation between categorical variables and discrete quantitative data with Fisher's exact test utilised when the cell size was less than five. If more than 20% of cells had a count less than five the variable categories were regrouped to enable analysis with Pearson chi‐square or Fisher's exact tests (Table 1, Table S1). A P value <0.05 was considered to be statistically significant. Phi coefficient (Φ) was used to assess the effect size of statistically significant results with Φ = 0.1 representing small effect size, Φ = 0.3 representing medium effect size and Φ ≥ 0.5 representing large effect size.

## Results

Responses to the survey were provided by 490 sonographers who met the inclusion criteria. Table 2 summarises the demographic characteristics of the survey respondents.

**Table 2:** Participant demographic characteristics (n = 490).

Characteristic	N (%)
Years of experience (regrouped)	
Less than 10 years	160 (32.7)
More than 10 years	330 (67.3)
Primary place of employment (regrouped)	
Public or private offering general ultrasound	375 (76.5)
Public or private offering specialised O&G ultrasound	104 (21.2)
Other (locum, currently not working)	11 (2.2)

O&G, obstetric and gynaecological.

### PCI site documentation

We assessed the extent to which the PCI site is included in departmental protocol for obstetric ultrasound examinations. Most sonographers indicated it is departmental protocol for the PCI to be documented at the time of the second trimester anatomy scan. Almost half of the respondents' departments include the PCI site in their departmental protocol at the 11‐ to 14‐week and 14‐ to 17‐week ultrasound and approximately one‐third of workplaces include the PCI site in their protocol after 22‐weeks gestation. A small percentage of responses indicated that it is not departmental protocol to document the PCI site. These findings are summarised in Table 3.

**Table 3:** Frequency of inclusion of the PCI site in departmental protocol (n = 490).

Ultrasound examination	N (%)
<11 weeks	14 (2.9)
11‐ to 14‐week ultrasound	213 (43.5)
Early second trimester anatomy scan (>14–17 weeks)	227 (46.3)
Second trimester anatomy scan (>17–22 weeks)	439 (89.6)
Ultrasound performed >22–28 weeks	152 (31.0)
Third trimester ultrasound	147 (30.0)
It is not departmental protocol to document the PCI	31 (6.3)

PCI, placental cord insertion.

Table 4 presents the frequency (%) of PCI site departmental protocol when comparing general and O&G ultrasound settings. There is no significant correlation between primary employment setting and departmental protocol at 14–17 weeks, the second trimester anatomy scan, ultrasound performed at 22–28 weeks and departments where it is not protocol to document the PCI. There is a significant correlation between primary employment setting and protocol to document the PCI site with a greater number of specialised O&G departments including the PCI site in their protocol at less than 11 weeks and 11–14 weeks. We also saw a significant association in third trimester with more general ultrasound practices including the PCI site as a requirement in their protocol when compared to O&G practices.

**Table 4:** Departmental protocol for PCI site documentation according to ultrasound setting.

Ultrasound examination	General setting, Percent (%)	Specialised O&G setting, Percent (%)	P	Φ
<11 weeks	2	6	0.041	0.041
11‐ to 14‐week ultrasound	41	52	0.045	0.057
Early second trimester anatomy scan (>14–17 weeks)	45	51	0.286	
Second trimester anatomy scan (>17–22 weeks)	90	91	0.6	
Ultrasound performed >22–28 weeks	31	28	0.516	
Third trimester ultrasound	32	20	0.017	0.021
It is not departmental protocol to document the PCI	7	3	0.108	

O&G, obstetric and gynaecological; PCI, placental cord insertion.

**Figure 7:**
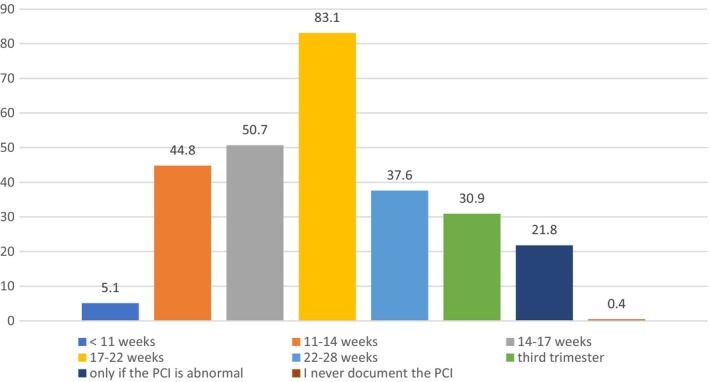
Sonographer documentation of the placental cord insertion site if not specified by departmental protocol (%).

Sonographers were asked to indicate the frequency in which they would document the PCI site during ultrasound examinations throughout pregnancy if not dictated by departmental protocol. The results are presented in Figure 7. We further analysed the data to establish whether there is any correlation between a sonographer's years of experience or their primary place of employment when documenting the PCI in the absence of departmental protocol (Table 5). Results show that sonographers with more than 10 years' experience are more likely to document the PCI site, despite normality, if it is not departmental protocol. In the absence of inclusion of the PCI site in the departmental protocol, our study shows no significant correlation between primary place of employment and documentation of the PCI site within all categories except third trimester.

**Table 5:** Sonographer documentation of the PCI if not specified in departmental protocol according to years of experience and employment setting.

Ultrasound examination	<10 years' experience, N (%)	>10 years' experience, N (%)	P	Φ	General, N (%)	Specialised O&G, N (%)	P	Φ
<11 weeks	5 (3.1)	19 (5.7)	0.205		12 (3.2)	11 (10.6)		0.002
11‐ to 14‐week ultrasound	63 (39.4)	149 (45.1)	0.226		159 (42.4)	48 (46.1)	0.494	
Early second trimester anatomy scan (14–17 weeks)	79 (49.4)	161 (49)	0.927		178 (47.5)	55 (53.4)	0.286	
Second trimester anatomy scan (17–22 weeks)	120 (75)	273 (83)	0.031		300 (80)	85 (83.3)	0.449	
Ultrasound performed 22–28 weeks	53 (33.3)	125 (37.9)	0.328		136 (36.4)	36 (34.6)	0.742	
Third trimester ultrasound	43 (26.9)	103 (31.3)	0.315		118 (31.5)	22 (21.3)	0.046	0.021
Only if abnormal	48 (30)	55 (16.7)	<0.001		80 (21.4)	22 (21.1)	0.958	
I never document the PCI	2 (1.2)	0		0.042	2 (0.5)	0		0.455

O&G, obstetric and gynaecological; PCI, placental cord insertion.

We compared the frequency in which the PCI site is documented in the departmental protocol, the sonographer's worksheet and the formal report (Table 6). Surprisingly, responses indicate departmental protocol dictates documenting the PCI site at the second trimester anatomy scan in 89.6% of responses yet the PCI site is only included in 60.5% of the formal reports. In fact, results show that the formal ultrasound report includes the PCI site less frequently than what the departmental protocol dictates and less often than it is documented on the sonographer's worksheet for all the ultrasound examinations listed in the survey.

**Table 6:** Comparison between frequency (%) of inclusion of PCI site in departmental protocol, sonographer's worksheet and formal report.

PCI documentation	Departmental protocol, (%)	Sonographer documentation if not departmental protocol, (%)	Formal report (%)
<11 weeks	2.9	5.1	1.5
11‐ to 14‐ week ultrasound	43.5	44.8	17.2
Early second trimester anatomy scan (14–17 weeks)	46.3	50.7	23
Second trimester anatomy scan (17–22 weeks)	89.6	83.1	60.5
Utrasound performed 22–28 weeks	31.0	37.6	17.6
Third trimester	30.0	30.9	16.1
Only if the PCI site is abnormal	–	21.8	42.7
It is never included	6.3	0.4	3.4

PCI, placental cord insertion.

We further analysed the data to determine whether there is any relationship between a sonographer's primary place of employment and the PCI site being included in the formal report – is the PCI site more likely to be included when issued by a department specialising in O&G ultrasound? Results showed the PCI site is recorded in the formal report more frequently in a specialised O&G setting (Table 7) with a statistically significant correlation at the second trimester anatomy scan (P = 0.008, Φ = 0.010).

**Table 7:** Inclusion of the PCI site in the formal report according to employment setting.

Ultrasound examination	General setting, (n = 375), N (%)	Specialised O&G, setting (n = 104), N (%)	P	Φ
<11 weeks	4 (1.1)	2 (1.9)	0.615	0.487
11‐ to 14‐week ultrasound	57 (15.2)	21 (20.2)	0.222	0.222
Early second trimester anatomy scan (14–17 weeks)	76 (20.3)	29 (27.9)	0.097	0.097
Second trimester anatomy scan (17–22 weeks)	205 (54.7)	72 (69.2)	0.008	0.008
Ultrasound performed 22–28 weeks	57 (15.2)	17 (16.3)	0.222	0.222
Third trimester ultrasound	54 (14.4)	17 (16.3)	0.621	0.621
Only if the PCI site is abnormal	160 (43.7)	36 (34.6)	0.159	0.159
It is never included	13 (3.5)	3 (2.9)	1.0	0.777

O&G, obstetric and gynaecological; PCI, placental cord insertion.

### Reassessment of the PCI site after the second trimester anatomy scan

Our study indicates the PCI site is regularly documented by sonographers prior to and at the second trimester anatomy scan (44.8% at the 11‐ to 14‐week ultrasound, 50.7% at 14‐ to 17‐week gestation and 83.1% at the second trimester anatomy scan); however, documentation decreases after the second trimester anatomy scan. Results suggest sonographers are more likely to reassess the PCI during ultrasound examinations after the second trimester anatomy scan if it was previously classified as marginal or velamentous (Table 8). Most respondents indicated they would assess the PCI site after 22 weeks if it were not documented at the second trimester anatomy scan.

**Table 8:** Frequency of sonographer assessment of the PCI site after 22 weeks according to PCI classification at the second trimester anatomy scan (n = 465).

PCI site at the second trimester anatomy scan	Assessment after 22 weeks, N (%)
Normal	187 (40.2)
Eccentric	342 (73.5)
Marginal	405 (87.1)
Velamentous	443 (95.3)
Not documented at the second trimester anatomy scan	374 (80.4)

PCI, placental cord insertion.

### Sonographer's opinions on the value of PCI documentation

**Figure 8:**
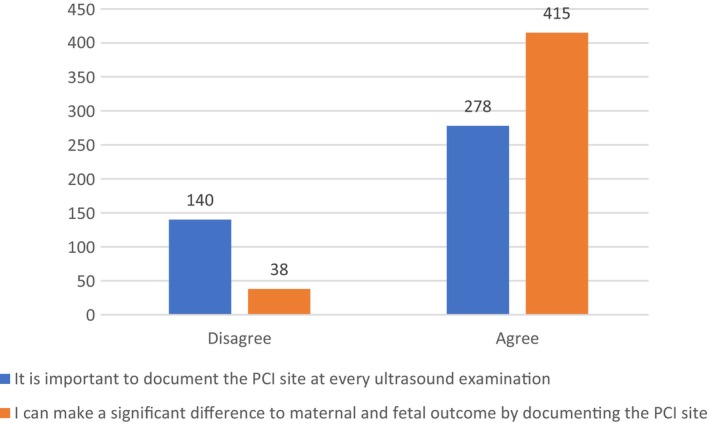
Sonographers' opinions on the value of placental cord insertiondocumentation (n).

More than half of the sonographers involved in our survey (59%) believe it is important to document the PCI site at every ultrasound with 29.7% of respondents disagreeing with this statement. Most respondents (87.6%) feel that documenting the PCI site can make a significant difference to maternal and fetal outcome with only 8% not agreeing (Figure 8). Results show no correlation between sonographer's opinions and their clinical experience and primary place of employment.

## Discussion

Internationally, there is wide variation regarding regulation of PCI documentation during antenatal ultrasound examinations. To our knowledge, this is the first study aimed at assessing the current practice of antenatal PCI documentation in Australia.

Our study shows a significant variation in departmental protocol for PCI site, the frequency of PCI documentation by sonographers and inclusion of the PCI site in the reporting specialist's formal report. It also reveals a reduction in documentation of the PCI after 22 weeks gestation which may be explained by increased difficulty in assessing the PCI in late third trimester, particularly when the placenta is posterior.^18^

### PCI site documentation

The American Institute of Ultrasound in Medicine (AIUM) and the International Society of Ultrasound in Obstetrics and Gynecology (ISUOG) recently updated their practice guidelines to include PCI as a standard component of the second trimester anatomy scan^45^, ^46^ with the AIUM also including PCI as a required component of ultrasound examinations between 12 weeks 0 days and 13 weeks 6 days gestation^47^ and in the third trimester ultrasound examination.^45^ Interestingly, the Australasian Society for Ultrasound in Medicine has not yet included the PCI site in their guidelines for the performance of first, second or third trimester ultrasound.^48^, ^49^, ^50^

A study by Sepulveda in 2006 showed screening for VCI in first trimester is feasible and allows for close monitoring for obstetric complications associated with VCI.^42^ Hasegawa *et al*.^44^ suggest PCI in the lower third of the uterus in the late first trimester is a predictor for vasa praevia. Our study shows specialised O&G practices include PCI site documentation at 11–14 weeks in their protocol more frequently than general ultrasound practices, suggesting more O&G practices are aware of the advantages of early documentation of the PCI site as outlined in the literature.^1^, ^31^, ^42^, ^44^ This is not surprising considering most O&G practices are operated by obstetricians who may value early knowledge of the PCI site, particularly if it is found to be abnormal.

Our results indicate that in the absence of departmental protocol, a sonographer's documentation of the PCI site on their provisional report varies according to the type of obstetric ultrasound being performed. Sonographers with more than 10 years' experience are more likely to document the PCI site at the second trimester anatomy scan in the absence of departmental protocol than those sonographers with less than 10 years' experience. We hypothesise that the more experienced group of sonographers are more likely to have been involved in cases in which knowledge of the PCI site has had influence on patient and/or fetal outcome.

In Australia, a formal ultrasound report is issued and authorised by a radiologist or sonologist at the completion of every ultrasound examination performed in an accredited ultrasound practice. In our experience, which incorporates over 20 years performing obstetric ultrasound examinations in public and private clinics in Western Australia and Queensland, there is great diversity in documentation of the PCI site in the formal ultrasound report. Our results show a large discrepancy between sonographer documentation of the PCI site on their worksheet and its inclusion in the formal ultrasound report, with sonographer documentation far outweighing that of the radiologist/sonologist. We theorise that sonographers are more likely to document the PCI site on their worksheet regardless of PCI normality, whilst radiologists/sonologists tend to report the PCI site only if abnormal. Our results also suggest the PCI site is recorded in the formal report more frequently by specialised O&G departments than general ultrasound departments for all examinations listed in the survey.

### Reassessment of the PCI site after the routine 17‐ to 22‐week ultrasound

Padula *et al*.^18^ showed PCI visualisation decreases with increasing gestational age with 98% PCI visualisation at 20–23 weeks gestation reducing to 72.2% at 35–38 weeks gestation. Their study also postulated that PCI documentation is more difficult when the placenta is posterior. Bosselmann and Mielke agree visualisation of the PCI is more challenging with advancing gestation.^2^ Several sonographers involved in our survey made comments that support these authors’ findings, for example ‘In the third trimester, sometimes it is difficult to be sure where the PCI is…’ and ‘it can be difficult to see with posterior placenta in late third trimester…’. Our results suggest the frequency of documentation of the PCI site in Australia decreases after 22 weeks gestation which may well be due to challenging visualisation of the PCI site later in pregnancy. However, Hasegawa *et al*. suggest difficulty in imaging the PCI site is an indicator for abnormal PCI.^8^ Therefore, if the PCI site is difficult to determine in the third trimester it may be due to poor visualisation of the PCI or it may indicate an abnormal PCI. This reiterates the importance of documenting the PCI in the first and second trimesters when visualisation of the PCI site is superior. It also supports documentation of the PCI site in the formal report in the first and second trimesters to assist sonographers who are evaluating the PCI for the first time in the third trimester.

Although our study suggests an overall reduction in PCI site documentation by sonographers after 22 weeks gestation, our results indicate most sonographers will reassess the PCI if it was classified as abnormal at the second trimester anatomy scan. Also of note is that most sonographers would assess the PCI site after 22 weeks if it had not been documented at the second trimester anatomy scan. A few sonographers commented that if the PCI site is not documented in the formal report of the second trimester anatomy scan, they would not assess the PCI at subsequent scans as they assume it was normal ‘…if it is not mentioned in the 17–22 week anatomy report, it is assumed to have been documented as normal’, ‘I assume it has been evaluated at the morphology scan’. In the interest of patient safety, we believe there should be no assumptions regarding the PCI site and if there is no previous formal documentation, we suggest the PCI site should be reassessed after 22 weeks.

Respondents indicated a high likelihood they would reassess the PCI site when the second trimester anatomy scan was not performed at their practice, even if the PCI had previously been documented as normal. Sonographers' comments included ‘If 17–22 week scan was performed elsewhere, I would reassess’. This demonstrates a need to feel confident about the PCI site, which in turn reflects the importance these sonographers place on PCI documentation.

### Sonographer's opinions on the value of PCI documentation

Fifty‐nine percent of sonographers involved in this survey chose the response It is very important to document the PCI at every ultrasound examination: ‘Our practice has found many abnormal PCIs since documenting at each scan, whether we have initially scanned the patient ourselves or as a follow up. Very important’. However, about a third of our respondents expressed opinions that this practice is not essential, for example ‘if it's very clearly been shown to be normal previously, I don't see that it's necessary to reassess at each scan…this doesn't offer any useful information and technically goes against ALARA’ (as low as reasonably achievable). This raises an important issue regarding ultrasound safety. Sonographers must keep the acoustic output and imaging duration as low as reasonably achievable whilst providing a medically diagnostic outcome.^51^, ^52^ Two authors have described a short imaging duration associated with PCI documentation. Nomiyama *et al*.^53^ recorded a mean time of 20 s to locate the PCI in the second trimester. Sepulveda *et al*.^35^ visualised 99.2% of PCI within the time allocated for a routine second and third trimester ultrasound. These authors’ findings suggest that the PCI site can be assessed without unduly prolonging the ultrasound examination, therefore adhering to the ALARA principle.

Our results suggest most respondents feel they can make a significant difference to maternal and fetal outcome by documenting the PCI site with no significant correlation between a sonographer's years of ultrasound experience or primary employment setting. We propose this indicates a sound knowledge amongst the surveyed sonographers in Australia of the potential adverse outcomes associated with abnormal PCI.

### Limitations

There are several limitations to this study. We received a low response rate for the survey. Out of a potential population size of 7353 sonographers, 534 responses were received (7.1% response rate). However, our sample size was sufficient to analyse data with a confidence interval of 96% and margin of error 5%. The sonographer survey was not pilot tested so we cannot be sure whether our questions were easy to interpret and whether options given for answers were comprehensive and relevant. The survey distribution was limited to one avenue and although recruitment was national through the ASAR, demographic data concerning location within Australia were not collected. Due to these limitations, survey responses received may not be representative of the entire profession in Australia.

## Conclusion

Our results support the current literature findings that there is variation regarding regulation of PCI documentation during antenatal ultrasound. Furthermore, it has shown diversity in inclusion of the PCI site in departmental protocols, sonographers' documentation on their worksheet and the formal ultrasound reports in Australia.

As discussed, the AIUM and ISUOG recently updated their practice guidelines regarding documentation of the PCI site during antenatal ultrasound examinations. This is an improved international recommendation we hope Australia will embrace. Unfortunately, even if the PCI site is a required component of the ultrasound examination, it does not ensure documentation in the formal report.

Assessment of the PCI at every ultrasound examination from 11 weeks gestation with inclusion of the PCI location in the formal ultrasound report, regardless of normality, would ensure the obstetric clinical team are aware of the PCI site. We propose establishing a national approach to PCI documentation in Australia to reduce maternal and fetal complications associated with abnormal PCI.

### Authorship statement

**Samantha Ward:** conceptualization; investigation; writing–original draft; methodology; visualization; formal analysis; resources; software. **Zhonghua Sun:** writing–review & editing; project administration; supervision; formal analysis. **Sharon Maresse:** writing–review & editing; project administration; supervision; formal analysis

## Funding

No funding information is provided.

## Conflict of interest

The authors declare no conflict of interest.

## Ethics approval

Your application was reviewed by the Curtin University Human Research Ethics Committee at their meeting on 07‐Sep‐2021. The review outcome is: Approved. Your proposal meets the requirements described in National Health and Medical Research Council's (NHMRC) National Statement on Ethical Conduct in Human Research (2007). Clinical trial registration numbers outline in ‘Sample Size’—sample size 490. Participant registration time frame outlined in ‘Study Design’—23/2/22 to 23/3/22. Participant consent outlined in ‘Study Design’—‘informed consent was obtained before participants commenced the survey’—Appendix S1.

## Supporting information


**Appendix S1.** Sonographer survey.

**Table S1.** Regrouping of variable categories.
