# Supplementary material for: Current practice of placental cord insertion documentation in Australia – A sonographer survey
Source: Australas J Ultrasound Med. 2023 Jul 26;26(3):157–68. doi: 10.1002/ajum.12360 (PMC10493351; doi:10.1002/ajum.12360)
Supplement: Supplementary file 1 — Appendix S1. Sonographer survey. [file AJUM-26-157-s002.docx]

S1. SUPPLEMENTAL INFORMATION – SONOGRAPHER SURVEY

Having read and understood the information provided, do you consent to participate in this questionnaire?

- Yes
- No – END OF SURVEY

Please answer each question to the best of your knowledge. Your responses are strictly confidential.

1. Do you perform Obstetric Ultrasound examinations?

- Yes
- No – END OF SURVEY

2. How long have you been practicing diagnostic medical ultrasound?

- < 1 year
- 1 – 5 years
- 5 – 10 years
- 10 – 15 years
- 15 – 20 years
- More than 20 years

3. What is your role as a Sonographer? (please select all that apply)

- Clinical Sonographer
- Head of Department
- Supervising Sonographer
- Tutor Sonographer
- Trainee Sonographer
- Other

If other, please specify

4. Which of the following best describes your primary place of employment

- Public sector offering General Ultrasound
- Private sector offering General Ultrasound
- Public sector offering Tertiary Level Obstetric/Gynaecological Ultrasound
- Private sector offering only Obstetric/Gynaecological Ultrasound
- Other

If other, please specify

5. What is your **primary** area of ultrasound practice?

- General
- Obstetrics
- Gynaecology
- Obstetrics and Gynaecology
- Breast
- Vascular
- Paediatrics
- Cardiac
- Other

If other, please specify

6. What hours do you work?

- Full time
- Part time
- Casual
- Other

If other, please specify

7. In your main workplace, is it **departmental protocol** for the placental cord insertion (PCI) site to be documented during: (please select all that apply)

- First trimester (< 11 weeks)
- 11-14 week ultrasound
- Early second trimester anatomy scan (14-17 weeks)
- Routine second trimester anatomy scan (17-22 weeks)
- Ultrasound performed after the routine anatomy scan (up to 28 weeks)
- Third trimester ultrasound
- It is not departmental protocol to document the PCI site at any gestation
- Other

If other, please specify

8. In your main workplace, when do you document the PCI site on your worksheet/preliminary sonographer’s report *even if it is not departmental protocol to do so*: (please select all that apply)

- First trimester (< 11 weeks)
- 11-14 week ultrasound
- Early second trimester anatomy scan (14-17 weeks)
- Routine second trimester anatomy scan (17-22 weeks)
- Ultrasound performed after the routine anatomy scan (up to 28 weeks)
- Third trimester ultrasound
- Only if the PCI is abnormal
- I never document the PCI site
- Other

If other, please specify

9. In your main workplace, when is the PCI site included in the Radiologist’s / Sonologist’s report? (please selec all that apply)

- First trimester (< 11 weeks)
- 11-14 week ultrasound
- Early second trimester anatomy scan (14-17 weeks)
- Routine second trimester anatomy scan (17-22 weeks)
- Second trimester ultrasound performed after the routine anatomy scan (up to 28 weeks)
- Third trimester ultrasound
- Only if the PCI is abnormal
- It is never included
- Other

If other, please specify

10. What classification/s of PCI does your main workplace use? (please select all that apply)

- Central (or normal)
- Eccentric
- Marginal
- Velamentous
- We don’t classify the PCI
- Other

If other, please specify

11. What criteria does your main workplace use to classify a PCI as being marginal?

- ≤ 10 mm from the closest placenta edge
- ≤ 20 mm from the closest placental edge
- ≤ 30 mm from the closest placental edge
- Other

If other, please specify

12. What images of the PCI do you document: (please select all that apply)

- PCI site, longitudinal plane
- PCI site, transverse image
- PCI site in 2 planes perpendicular to each other
- I don’t document the placental cord insertion site
- Other

If other, please specify

13. When documenting the PCI site which of the following ***measurements*** do you record?

- I always measure the distance of the PCI site from the closest placental edge, even if the PCI is normal
- I only measure the distance of the PCI site from the closest placental edge if the PCI site is abnormal
- I never measure the distance of the PCI site from the closest placental edge
- I don’t document the PCI
- Other

If other, please specify

14. If the PCI is shown to be velamentous, which of the following would you routinely document? (please select all that apply)

- The position of the velamentous cord insertion (upper, mid or lower uterus, right/left lateral etc)
- The length of the unprotected vessels
- The path of the unprotected vessels
- Assessment for associated vasa praevia
- Other

If other, please specify

15. If the PCI site is documented as normal (central) at the 17-22 week anatomy scan, do you assess it again during subsequent ultrasounds performed?

- Yes
- No
- Other

If other, please specify

16. If the PCI is documented as eccentric at the 17-22 week anatomy scan, do you assess it again during subsequent ultrasounds performed?

- Yes
- No
- Other

If other, please specify

17. If the PCI is documented as marginal at the 17-22 week anatomy scan, do you assess it again during subsequent ultrasounds performed?

- Yes
- No
- Other

If other, please specify

18. If the PCI is documented as velamentous at the 17-22 week anatomy scan, do you assess it again during subsequent ultrasounds performed?

- Yes
- No
- Other

If other, please specify

19. If the PCI ***has not been documented*** at the 17-22 week anatomy scan, do you assess it again during subsequent ultrasounds performed?

- Yes
- No
- Other

If other, please specify

20. Please select your level of agreement with the following statements:

|  | Strongly Disagree | Somewhat Disagree | Neither agree nor disagree | Somewhat Agree | Strongly Agree | Uncertain |
| --- | --- | --- | --- | --- | --- | --- |
| It is important to document the PCI at every ultrasound examination performed |  |  |  |  |  |  |
| I can make a significant difference to a patient’s outcome by documenting the PCI |  |  |  |  |  |  |

21. Please feel free to add any comments relevant to this survey.

Thank you for taking the time to participate in this survey.
