# Supplementary material for: Current practice of placental cord insertion documentation in Australia – A sonographer survey
Source: Australas J Ultrasound Med. 2023 Jul 26;26(3):157–68. doi: 10.1002/ajum.12360 (PMC10493351; doi:10.1002/ajum.12360)
Supplement: Supplementary file 2 — Table S1. Regrouping of variable categories. [file AJUM-26-157-s001.docx]

S2. SUPPLEMENTAL INFORMATION

**Table 1.** Regrouping of variable categories

| Variable | Categories | Regrouped categories |
| --- | --- | --- |
| Years of experience | - < 1 year - 1 – 5 years - 5 – 10 years - 10 – 15 years - 15 – 20 years - > 20 years | - Less than 10 years’ experience - More than 10 years’ experience |
| Primary place of employment | - Public sector offering general ultrasound - Private sector offering general ultrasound - Public sector offering tertiary level O&G† ultrasound - Private sector offering only O&G† ultrasound | - Public or private offering general ultrasound - Public or private offering specialised O&G† ultrasound |
| “It is important to document the PCI site at every ultrasound examination” | - Strongly disagree - Somewhat disagree - Neither agree nor disagree - Somewhat agree - Strongly agree - Uncertain | - Disagree - Neither agree nor disagree - Agree |
| “I can make a significant difference to maternal and fetal outcome by documenting the PCI site” | - Strongly disagree - Somewhat disagree - Neither agree nor disagree - Somewhat agree - Strongly agree - Uncertain | - Disagree - Neither agree nor disagree - Agree |

†Obstetric and gynaecological.
